# Study of Bending Strength Detection Method for SMC Composites Based on Laser-Induced Breakdown Spectroscopy

**DOI:** 10.3390/ma19091714

**Published:** 2026-04-23

**Authors:** Hongbo Wang, Mengke Gao, Zhe Qiao, Junchen Li, Xuhui Cui, Xilin Wang

**Affiliations:** 1 State Key Laboratory of Smart Power Distribution Equipment and System, State Grid Jibei Electric Power Company Limited, Beijing 100054, China; 18533566257@163.com (H.W.); 15933086796@163.com (M.G.); qiaozhe2021@163.com (Z.Q.); 2 State Grid Jibei Electric Power Company Limited Smart Distribution Network Center, Qinghuangdao 066100, China; 3 Tsinghua Shenzhen International Graduate School, Tsinghua University, Shenzhen 518000, China; lijunchen25@mails.tsinghua.edu.cn (J.L.); xhthu@163.com (X.C.)

**Keywords:** sheet molding compound, Laser-Induced Breakdown Spectroscopy, bending strength, thermal aging

## Abstract

Electric energy metering cabinets serve as critical nodes in power grid operations, providing essential protection for key components in distribution networks. Under environmental stressors, the non-metallic casings of electric energy metering cabinets are susceptible to aging-induced performance degradation, which may result in electrical safety hazards. However, rapid and precise methods for evaluating the performance of these non-metallic casings are still lacking. Laser-Induced Breakdown Spectroscopy (LIBS), capable of rapid multi-element detection with non-contact analytical advantages, was employed in this study. Thermal aging experiments were conducted to investigate the performance degradation mechanisms of sheet molding compound (SMC)—a representative non-metallic cabinet material. The research analyzed time-dependent trends in material performance and microstructural evolution during aging. By integrating LIBS with multi-analytical techniques, this study further explored the feasibility of quantitatively evaluating the bending strength of thermally aged SMC, which has rarely been reported in previous studies. Based on LIBS spectral data, bending strength characterization revealed its attenuation patterns with aging duration. The relationships between bending strength and plasma temperature, as well as the characteristic line intensity ratios of K, Al, and Ca, were systematically examined. A multivariate linear regression model incorporating these key variables was subsequently developed, yielding a high coefficient of determination (R^2^ = 0.9657) between the predicted and measured bending strength values. This model represents a promising initial step, but further validation with a larger dataset is necessary to enhance its reliability and generalizability.

## 1. Introduction

As an indispensable component of power systems, the electric energy metering cabinet plays a vital role in protecting key devices such as smart energy meters, data acquisition terminals, and circuit breakers. As the interface between power grids and end-users, it also serves as an important IoT sensing node for the refined operation and maintenance of distribution transformer areas. With the rapid development of smart grids, the quality and reliability of electric energy metering cabinets have become increasingly important for power grid security, service efficiency, and socio-economic stability [1,2].

Among the materials used in these cabinets, Sheet Molding Compound (SMC) has become a representative non-metallic material for outdoor electrical equipment due to its excellent overall performance. SMC is typically composed of an unsaturated polyester resin matrix reinforced with chopped glass fibers or carbon fibers, together with fillers, thickeners, low-shrinkage additives, and other auxiliaries [3,4,5]. Owing to its good processability, high mechanical strength, electrical insulation, thermal stability, and chemical resistance, SMC has been widely used in the automotive, aerospace, and electrical industries [6,7]. However, under long-term outdoor service conditions, SMC is inevitably exposed to ultraviolet radiation, thermal cycling, and humidity fluctuations, which gradually degrade its mechanical and insulating properties. In particular, the reduction in bending strength directly affects the structural integrity and operational safety of electric energy metering cabinets. Conventional methods such as three-point bending tests are destructive and require complex specimen preparation, making them unsuitable for rapid and in situ condition assessment.

Laser-Induced Breakdown Spectroscopy (LIBS) is a rapid compositional analysis technique based on atomic emission spectroscopy. When a high-energy pulsed laser is focused on the sample surface, localized ablation generates a transient plasma plume, and qualitative or quantitative information can be obtained from the characteristic radiation emitted during plasma relaxation [8,9]. Once the laser energy density exceeds the material breakdown threshold, optical breakdown is triggered, followed by ionization and plasma formation through electron avalanche processes [10,11,12,13]. Owing to its rapid response, multi-element detection capability, minimal sample preparation, and non-contact or micro-destructive nature, LIBS has shown broad application potential in environmental monitoring, materials science, geological surveys, biomedicine, and space exploration [14,15].

Recent studies have extended LIBS from elemental analysis to material state and performance characterization. Researchers from the Institute of Modern Physics, Chinese Academy of Sciences [16] investigated the effects of powder packing density and thickness on LIBS signals for online monitoring of nuclear fuel powders. Do et al. [17] used LIBS for gas density measurement in a supersonic crossflow cavity flameholder and established correlations between plasma energy and gas density. Sivakumar et al. [18] examined the influence of packing density on LIBS measurements and showed that optimizing the laser-spot-to-particle-size ratio could reduce spectral fluctuations. In the field of electrical insulating materials, Chen et al. [19] correlated LIBS spectral features with the tracking resistance grades of silicone rubber. These studies indicate that LIBS can provide information related not only to elemental composition but also to material state and functional performance.

However, previous studies have mainly focused on classification analysis or indirect performance evaluation, while quantitative characterization of the bending strength of thermally aged SMC based on LIBS has rarely been reported. In our previous work [1], LIBS was applied to classification-related research on electrical insulating materials, whereas the present study further advances this direction by targeting quantitative mechanical characterization. To address this gap, this study combines LIBS with ATR-FTIR, SEM, and ICP analyses to investigate the thermal aging behavior of SMC and to establish quantitative relationships between spectral features and bending strength. Previously, no relevant studies have used LIBS to detect the bending strength of SMC. In this study, we not only propose using plasma temperature and characteristic spectral line intensity ratios to characterize bending strength, but also introduce a multivariate linear regression model, which significantly improves prediction performance. Compared with conventional destructive testing, the proposed method offers minimal sample preparation, low damage, and rapid analysis, providing a feasible route for non-destructive evaluation of SMC enclosure performance.

## 2. Materials and Methods

### 2.1. Experimental Devices

**Figure 1:**
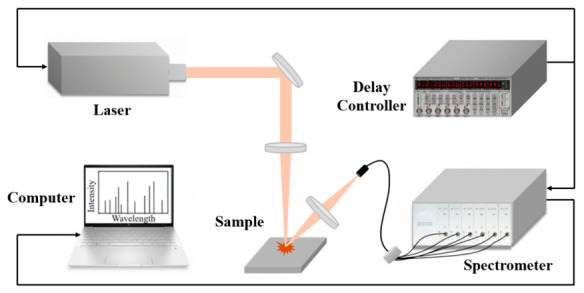
Schematic of LIBS device.

Figure 1 illustrates the hardware configuration of the Laser-Induced Breakdown Spectroscopy (LIBS) system, comprising five core components: a pulsed laser source, optical transmission assembly, spectral acquisition module, timing control unit, and data processing terminal. The operational workflow can be summarized as follows: Initially, a pulsed laser emits high-energy beams (triggered by external signals) that are redirected via dielectric mirrors and focused onto the sample surface through a plano-convex lens, generating a 50 μm diameter focal spot with peak power density reaching gigawatt-per-square-centimeter (GW/cm^2^) magnitudes. When the laser energy exceeds the sample’s breakdown threshold, sequential ablation–vaporization–ionization processes occur, creating transient plasma. During plasma cooling, element-specific emission lines are spatially resolved through a coaxial short-focal-length collection system, with optical signals transmitted via fiber optics to a high-resolution spectrometer. Acquired spectral data are transferred via USB to a computer for analysis, with the entire system synchronized by a digital delay generator.

### 2.2. Surface Functional Group Analysis of Aged SMC Samples

Prolonged exposure of SMC materials to elevated temperatures induces structural aging and consequent performance degradation. Experimental observations revealed molecular chain scission in the resin matrix and modifying additives under thermal stress, manifesting as surface darkening, diminished gloss, and powdering phenomena. Progressive aging weakens the interfacial bonding between fibers and the matrix, leading to significant deterioration in mechanical properties such as bending strength and impact toughness. These findings underscore the necessity of accelerated aging studies to elucidate microstructural degradation mechanisms critical for material durability assessment.

**Figure 2:**
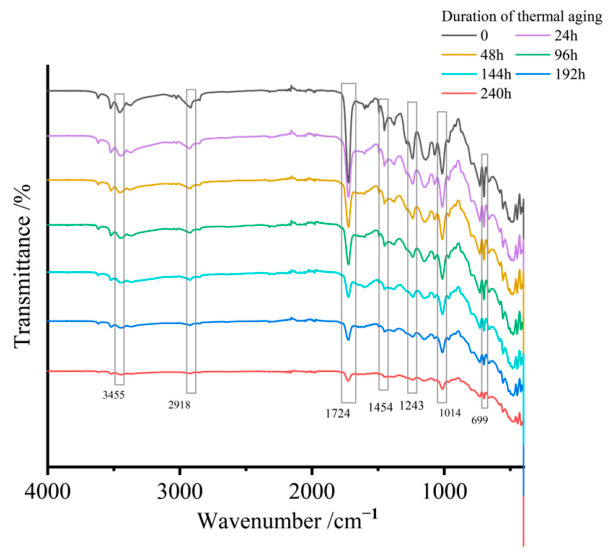
ATR spectra of SMC samples with different aging durations.

Seven SMC panels from the same manufacturer underwent accelerated thermal aging at 230 °C (±2 °C) with exposure durations of 0 h, 24 h, 48 h, 96 h, 144 h, 192 h, and 240 h. Attenuated total reflection (ATR) Fourier-transform infrared spectroscopy was used to characterize surface functional group evolution across aging intervals, with full-spectrum profiles shown in Figure 2.

The ATR spectra revealed characteristic absorption bands corresponding to hydroxyl (-OH) stretching vibrations at 3455 cm^−1^, aliphatic C-H stretching at 2918 cm^−1^, and carbonyl (-C=O) vibrations at 1724 cm^−1^. Additional diagnostic peaks included carboxyl (-COOH) stretching at 1454 cm^−1^, methyl (-CH_3_) rocking modes at 1243 cm^−1^, siloxane Si-O-Si asymmetric stretching at 1014 cm^−1^, and Si-O bending vibrations at 699 cm^−1^.

Comparative analysis demonstrated progressive attenuation of characteristic peak intensities with aging duration. The carbonyl (-C=O), methyl (-CH_3_), and siloxane (Si-O-Si) groups showed particularly pronounced degradation. Critical functional groups, including hydroxyl (-OH) and C-H bonds exhibited near-complete signal depletion, approaching baseline noise levels.

The utilization of thickeners plays a critical role in enhancing the mechanical properties of SMC materials [20]. At the molecular scale, the synergistic interaction between magnesium oxide (MgO) thickeners and unsaturated polyester resin operates through dual reaction pathways. The first involves acid–base neutralization between terminal carboxylic acid groups (-COOH) in resin chains and MgO particles, generating linear magnesium carboxylate salts. This reaction significantly increases the polymer system’s average molecular weight, creating three-dimensional networks through physical chain entanglement—the primary mechanism for viscosity enhancement. Simultaneously, a secondary chemical coordination mechanism occurs between carbonyl groups (-C=O) in resin molecule and Mg^2+^ ions, forming crosslinkable metal coordination complexes. These coordination bonds not only amplify intermolecular forces but also establish supplementary three-dimensional networks, synergistically enhancing the viscoelastic properties of SMC composites.

**Figure 3:**
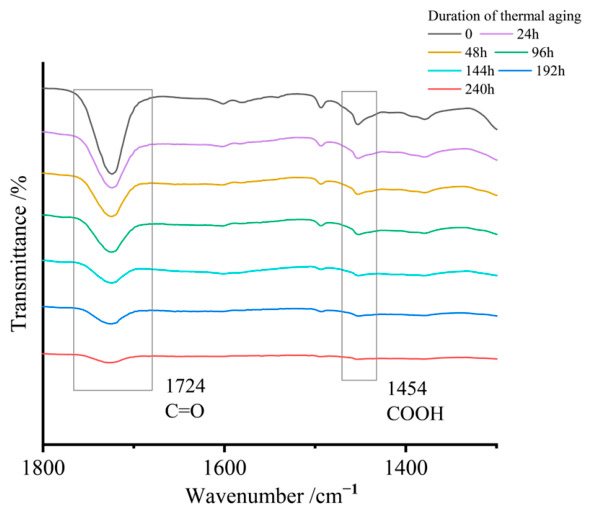
Characteristic peaks related to the thickening reaction.

**Figure 4:**
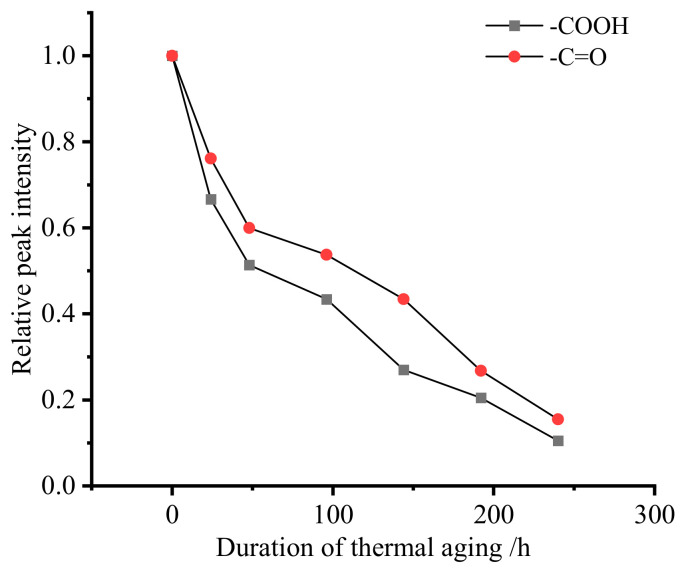
The attenuation of characteristic peaks of thickening products with aging time.

The ATR spectra in Figure 3 demonstrate progressive attenuation of characteristic peaks during thermal aging. The carboxylic acid (-COOH) stretching vibration at 1454 cm^−1^ exhibits an 89.48% intensity reduction, while the carbonyl (-C=O) absorption at 1724 cm^−1^ shows an 84.50% intensity loss. The nonlinear decay profile, detailed in Figure 4, reveals a two-stage degradation process: rapid initial intensity decline followed by a slowed decomposition rate. This behavior aligns with the thermal decomposition kinetics of magnesium carboxylates and the progressive destabilization of metal–carbonyl coordination networks.

These molecular-level structural changes drive critical macroscopic performance degradation. The decomposition of carboxylate salts shifts the molecular weight distribution toward lower ranges, weakening the resin matrix’s load-bearing capacity. Concurrently, the collapse of coordination networks reduces the interface bonding strength between the resin and glass fibers. These cumulative effects ultimately lead to the deterioration of critical mechanical performance metrics, including flexural modulus and impact toughness.

Coupling agents exert a pivotal influence on the mechanical properties of SMC composites. As a core component for interface optimization, silane coupling agents establish chemical bridging networks between the resin matrix and inorganic fillers. The alkoxy groups at their molecular terminals form covalent bonds with filler surface hydroxyls via hydrolysis, while reactive functional groups at the opposite ends chemically graft onto the resin matrix. This amphiphilic molecular design significantly enhances organic–inorganic phase compatibility, improving stress transfer efficiency across heterogeneous interfaces.

**Figure 5:**
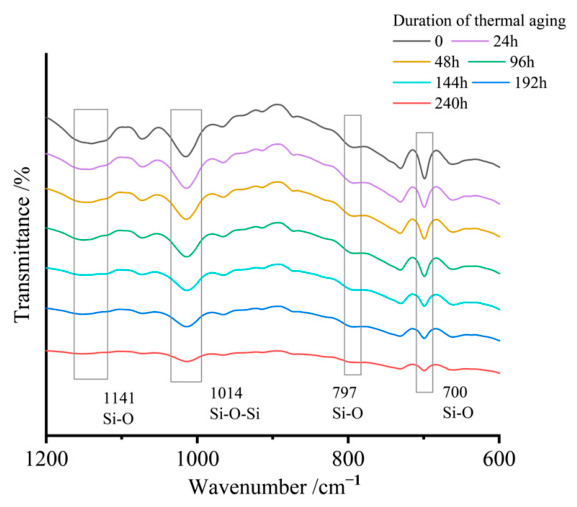
Characteristic peaks related to the coupling reaction.

The degradation dynamics of these interfacial structures are captured in Figure 5, which tracks characteristic silane-derived chemical bond signatures during thermal aging. Experimental data reveal progressive attenuation of these peaks, which provides clear evidence of thermally induced siloxane bond cleavage and interfacial bridge structure disintegration.

Mechanistically, the declining Si-O bond density weakens filler–matrix interfacial adhesion, reducing shear stress transfer efficiency. Under mechanical loading, these compromised interfaces become preferential stress concentration zones, with crack propagation preferentially following filler–resin boundaries. Consequently, the fiber reinforcement phase loses its load-bearing capacity, directly correlating with the bending strength loss in aged specimens.

### 2.3. Microstructural Morphology Analysis of Aged SMC Samples

**Figure 6:**
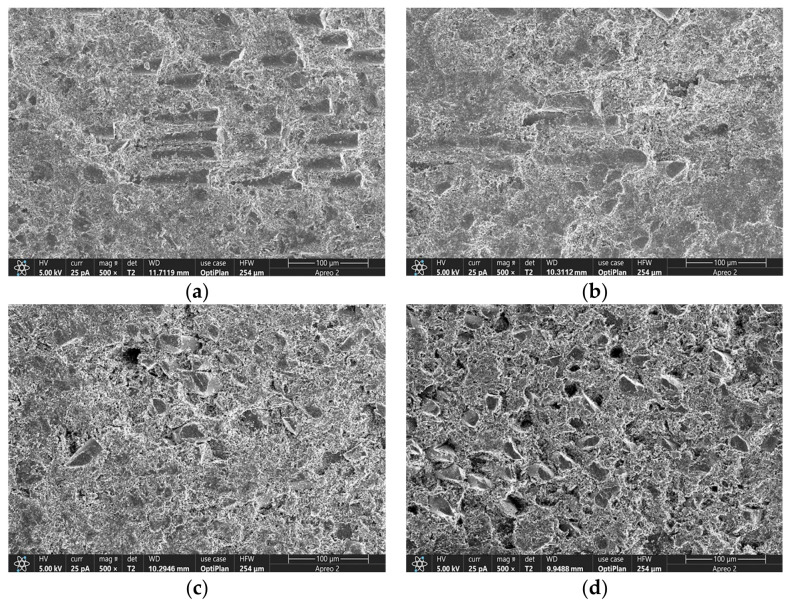
Microstructural morphology of SMC samples at different aging times: (**a**) 0 h; (**b**) 48 h; (**c**) 144 h; (**d**) 240 h.

Scanning electron microscopy (SEM) was employed to characterize the fracture surfaces of SMC materials before and after thermal aging, as illustrated in Figure 6, to investigate the degradation mechanisms of their microstructures. Figure 6a displays the pristine sample’s morphology, revealing a dense, homogeneous resin matrix with ideal interfacial bonding to the glass fiber reinforcement phase. The fibers exhibit well-aligned parallel laminar arrangements, and the fiber–matrix interfacial transition zones show no discernible defects—a structural configuration that ensures efficient load transfer across heterogeneous components.

Significant morphological alterations emerge after 230 °C thermal aging. As shown in Figure 6b (48 h aging), the resin matrix develops partial melting and re-solidification features, accompanied by localized microvoids induced by thermal expansion. The reinforcement fibers exhibit reduced contour definition and increased inter-fiber spacing, attributed to volatiles escaping from the thermally decomposed resin matrix and interfacial debonding.

Extended aging intensifies structural damage, as shown in Figure 6c,d. The resin matrix develops axial crack propagation along fiber orientations and forms honeycomb-like porous structures due to thermal stress relaxation. Glass fibers exhibit topological distortion with irregular fracture morphologies. These microstructural degradations reduce effective load-bearing cross-sections and promote preferential crack propagation paths under stress, directly correlating with the reduction in bending strength.

### 2.4. Elemental Concentration Evolution and Material Degradation Correlation

**Figure 7:**
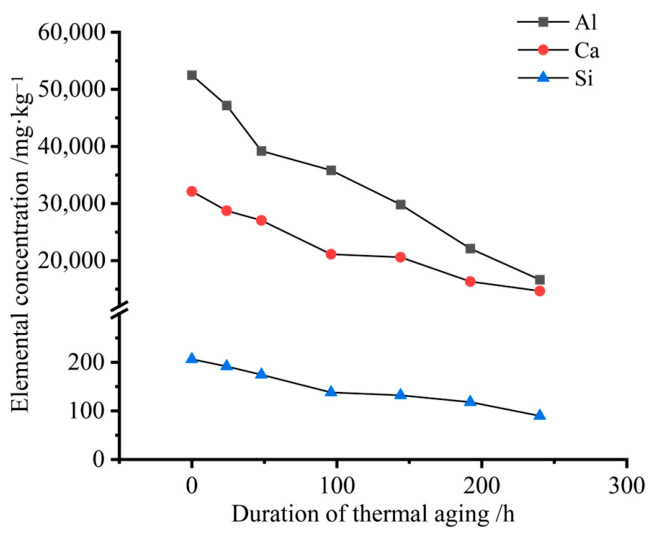
Elemental content variation in SMC samples with aging time.

For ICP-OES analysis, the SMC samples at different aging stages were first crushed and ground into fine powder. A certain amount of powder was weighed and subjected to acid digestion, and the resulting solution was diluted to a constant volume with deionized water before elemental analysis. The concentrations of Al, Ca, and Si were then determined by ICP-OES. Quantitative analysis via inductively coupled plasma optical emission spectroscopy (ICP-OES) revealed time-dependent depletion patterns of aluminum (Al), calcium (Ca), and silicon (Si) in thermally aged SMC materials, as shown in Figure 7. Aluminum, primarily derived from flame-retardant aluminum trihydroxide (ATH) and thickener alumina (Al_2_O_3_) particles, exhibited the most severe depletion (68.27% loss after 240 h of aging). Calcium, originating from calcium carbonate (CaCO_3_) fillers, showed 54.31% mass reduction, serving as a critical indicator of inorganic filler retention. Silicon content, from siloxane structures and glass fibers, decreased by 56.56%, reflecting interfacial degradation.

The differential depletion patterns correlate with distinct thermal degradation mechanisms. Calcium loss predominantly resulted from physical adsorption failure at filler–matrix interfaces, where thermal stress-induced debonding reduced interfacial adhesion strength, enabling CaCO_3_ particle escape through microcrack networks. The depletion of aluminum element was attributed to the partial thermal decomposition of aluminum trihydroxide (ATH) flame retardant initiated at the experimental temperature of 230 °C, coupled with the volatilization of resultant decomposition byproducts. Silicon reduction stemmed from dual pathways: hydrolytic cleavage of silane coupling agents’ Si-O bonds and partial dissociation of glass fibers’ surface siloxane networks.

The aforementioned analysis demonstrates that the depletion of different elements directly reflects material performance degradation: the progressive reduction of calcium carbonate leads to the weakening of SMC materials’ rigid supporting framework, the loss of aluminum hydroxide diminishes the effectiveness of the flame retardant system, and the decline in silicon content indicates compromised interfacial bonding performance, resulting in reduced fiber–resin interfacial shear strength. This enables the establishment of mapping relationships between elemental concentrations and material performance parameters, thereby achieving indirect assessment of material condition.

## 3. Results and Discussion

### 3.1. Bending Strength Testing

**Figure 8:**
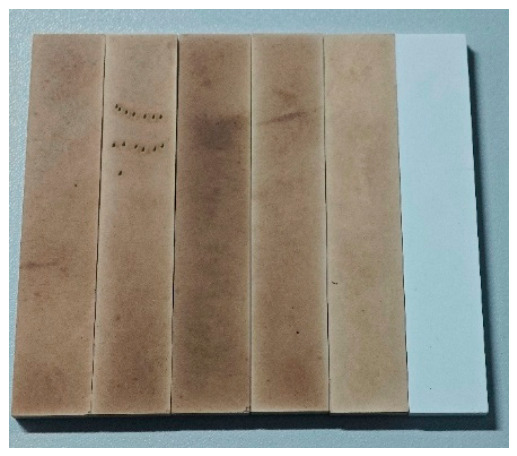
SMC strip specimens with different aging durations.

Six groups of SMC samples were prepared by machining the material into standard rectangular specimens with a length of 80 mm, width of (15 ± 0.5) mm, and thickness of (4 ± 0.2) mm for thermal-oxidative aging experiments. The thermal aging process was carried out in air using a drying oven with direct temperature control. The aging temperature was set to 230 °C, which was selected to accelerate the thermal-oxidative degradation process within a reasonable experimental time while preserving the basic macroscopic integrity of the samples for subsequent characterization and testing, with aging durations of 0 h (untreated control group), 48 h, 96 h, 144 h, 192 h, and 240 h. For each aging duration, five independent specimens were tested, and the average bending strength value was used for subsequent analysis. As shown in Figure 8, the color of specimens subjected to different aging durations exhibited a distinct transitional trend: the surface progressively transitioned from an initial white to a dark yellowish-brown hue as aging time increased, a phenomenon attributed to thermal oxidation reactions in the polymer matrix. Additionally, microcrack initiation was observed on specimen sidewalls, caused by the mismatch in thermal expansion coefficients between the resin and fibers.

**Figure 9:**
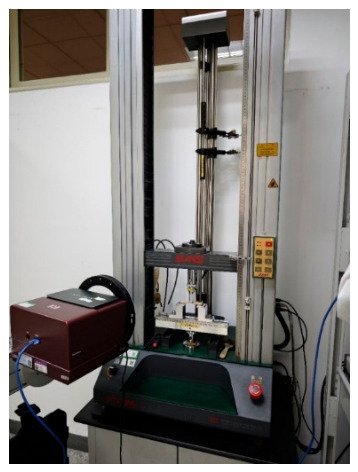
Three-point bending test.

Subsequent bending strength testing was performed using an electronic universal testing machine. As illustrated in Figure 9, a support span of 60 mm and a test speed of 10 mm/min were selected, with bending strength calculated via the following formula:

(1) $$\sigma_{f}=\frac{3P\cdot l}{2b\cdot h^{2}}$$

In the formula, $\sigma_{f}$ represents the bending strength in megapascals (MPa); P denotes the failure load (or maximum load) in newtons (N); l is the span in millimeters (mm); h is the specimen thickness in millimeters (mm); and b is the specimen width in millimeters (mm).

**Figure 10:**
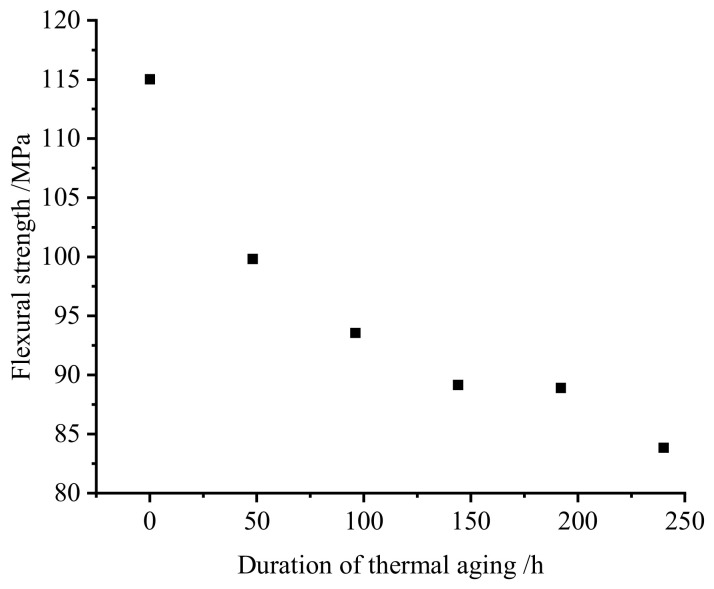
Variation in bending strength with aging time.

The bending performance data of each sample group obtained through standard experimental methods are detailed in Table 1, with Figure 10 visually demonstrating the evolution of material bending strength with thermal aging duration. Experimental results reveal an approximately exponential decay trend in the bending strength of SMC composites as aging time increases, a phenomenon closely associated with the material’s cumulative thermal damage characteristics.

**Figure 11:**
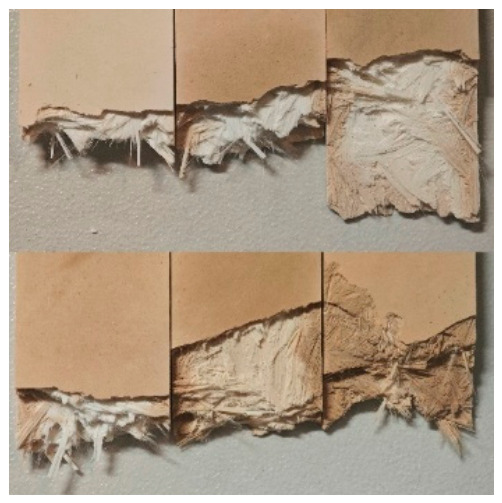
Fracture surfaces of SMC samples with different aging durations.

**Figure 12:**
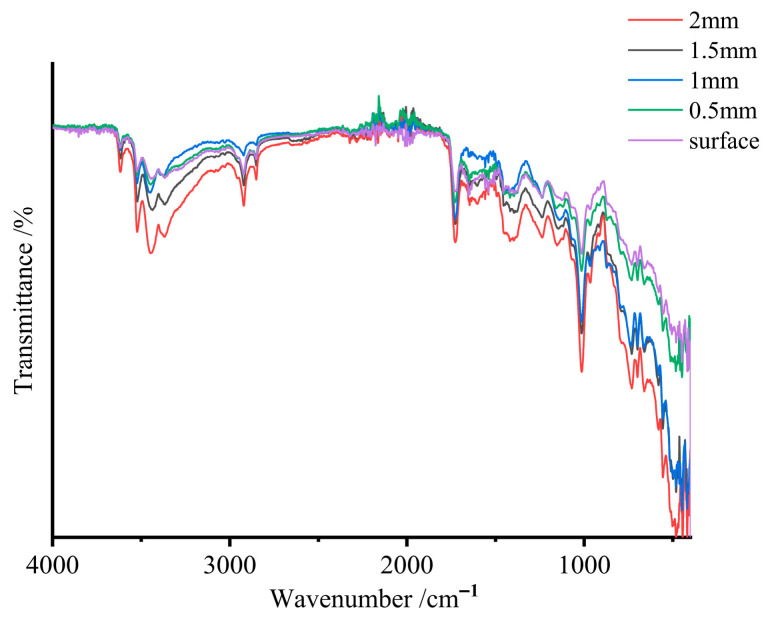
ATR spectra at different depths from the sample surface.

Observation of fracture surfaces from SMC samples at different aging stages, as shown in Figure 11, indicates that the region nearest to the surface undergoes initial discoloration. With prolonged aging, the yellowing progressively propagates inward, with near-complete internal discoloration observed in the longest-aged specimens. Sampling at varying depths from the surface followed by ATR testing (Figure 12) quantitatively evaluates this aging gradient.

Analysis of characteristic peak intensity variations demonstrates progressive enhancement with increasing depth from the surface, confirming reduced aging severity in the material’s interior. The shallowest region (within 0.5 mm depth) exhibits minimal peak intensities and maximal aging degradation, while the central region (2 mm depth) maintains significantly lower aging levels compared to surface areas.

These findings establish that thermal aging in SMC propagates from the surface to the core, with maximum degradation occurring in subsurface regions (0.1–0.5 mm depth). These mechanically compromised zones, exhibiting the poorest performance, preferentially initiate failure under external loads and dominantly govern the material’s global mechanical strength. The LIBS technique’s characteristic sampling depth (100–200 μm) precisely interrogates these critical subsurface regions, enabling reliable correlation between LIBS spectral data and bulk mechanical properties of SMC materials.

### 3.2. Relationship Between LIBS Plasma Parameters and Bending Strength

The six groups of SMC samples were analyzed using an LIBS system with the following parameters: laser pulse repetition rate of 2 Hz, incident angle perpendicular to the sample surface, spot size set to 50 μm, laser energy of 45 mJ, laser wavelength of 1064 nm, pulse duration of ≤9 ns, delay time of 1.5 μs, and 20 consecutive laser pulses. The emission spectra were collected by a six-channel spectrometer covering the wavelength range of 200–640 nm, with a spectral resolution of 0.09–0.13 nm. To minimize analytical errors, the spectral data were averaged.

Plasma temperature, a critical characteristic parameter in LIBS analysis, reflects the excitation state and energy distribution of the plasma, indicating the efficiency of energy transfer among particles and the degree of ionization equilibrium [21,22,23]. Assuming the laser-generated plasma satisfies the local thermodynamic equilibrium (LTE) condition, the Boltzmann plot method was employed to calculate plasma temperature. In LIBS analysis, the LTE assumption is commonly adopted for plasma temperature determination, and its validity can be evaluated using the McWhirter criterion. The specific procedure involves selecting spectral lines from different excitation states of the same element (atomic or ionic) to establish quantitative relationships between line intensities and energy level distributions. For a specific emission line corresponding to a transition from upper energy level k to lower level i, the intensity $I_{ki}$ follows the relationship:

(2) $$\ln\left(\frac{I_{ki}\lambda_{ki}}{g_{k}A_{ki}}\right)=-\frac{E_{k}}{k_{B}T_{e}}+C$$

Here, $\lambda_{ki}$ represents the spectral line wavelength; $g_{k}$, $E_{k}$, and $A_{ki}$ denote the statistical weight, excitation energy, and transition probability of the upper energy level, respectively; $k_{B}=1.3806\times 10^{-23}\;J/K$ is the Boltzmann constant; $T_{e}$ is the plasma temperature; and C is a constant term.

A Boltzmann plot was subsequently constructed with excitation energy $E_{k}$ as the abscissa and $\ln\left(I_{ki}\lambda_{ki}/g_{k}A_{ki}\right)$ as the ordinate. By calculating and substituting the ordinate values of multiple distinct spectral lines from the same element, the plasma temperature $T_{e}$ was determined from the slope of the fitted line, $-E_{k}/k_{B}T_{e}$.

This study selected multiple calcium (Ca) spectral lines for Boltzmann plot construction. The chosen spectral lines and their associated parameters are listed in Table 2, with data sourced from the NIST database.

**Figure 13:**
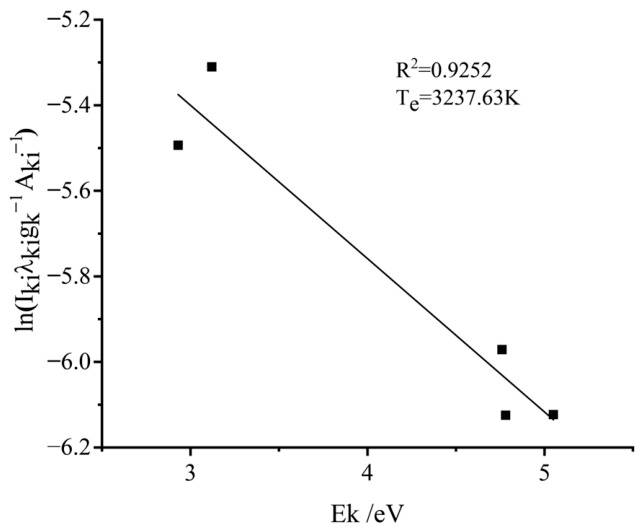
Boltzmann plot for Sample 1.

Taking Sample 1 as an example, the Boltzmann plot is shown in Figure 13. The goodness-of-fit (R^2^) for the linear regression is 0.9252, with a slope value of −0.3584, yielding a calculated plasma temperature of $T_{e}=3237.63\;K$. Note that the conversion factor 1 eV = 1.602 × 10^−19^ J was applied in the calculation.

**Figure 14:**
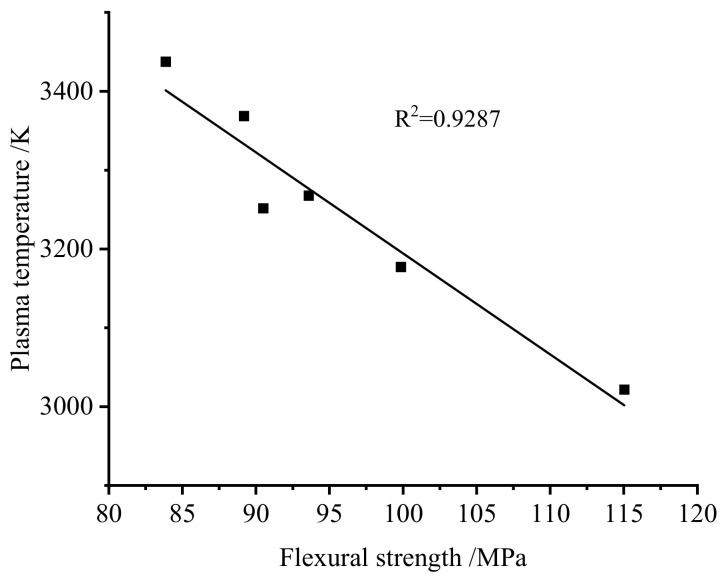
Relationship between plasma temperature and bending strength.

The plasma temperature of each sample was calculated using the same method, and the relationship between plasma temperature and bending strength is shown in Figure 14. Experimental results demonstrate a significant linear correlation between plasma temperature and SMC bending strength, with a goodness-of-fit R^2^ value reaching 0.9287.

Materials with higher bending strength generally possess a more intact microstructure, characterized by a denser resin crosslinking network and stronger fiber–resin interfacial bonding. Under laser irradiation, such a structure is less susceptible to localized damage and rapid material removal. As a result, a larger proportion of the incident laser energy is likely consumed by bond dissociation, heat conduction, and internal energy dissipation within the material, rather than being converted into plasma excitation and ionization. Consequently, both the ablation efficiency and plasma formation efficiency decrease, leading to a lower plasma temperature. In contrast, thermally aged SMC with lower bending strength typically exhibits matrix degradation, interfacial debonding, and a looser near-surface structure, which facilitates laser-induced ablation and increases the fraction of energy contributing to plasma generation. While the current explanation provides a reasonable physical framework for the observed inverse correlation, it should be noted that, due to experimental limitations, factors such as thermal conductivity and bond energy are not directly available in this study. Therefore, this hypothesis can be regarded as an avenue for further validation in future research. This provides a reasonable physical explanation for the experimentally observed inverse correlation between plasma temperature and bending strength.

### 3.3. Relationship Between LIBS Spectral Line Intensity Ratio and Bending Strength

**Figure 15:**
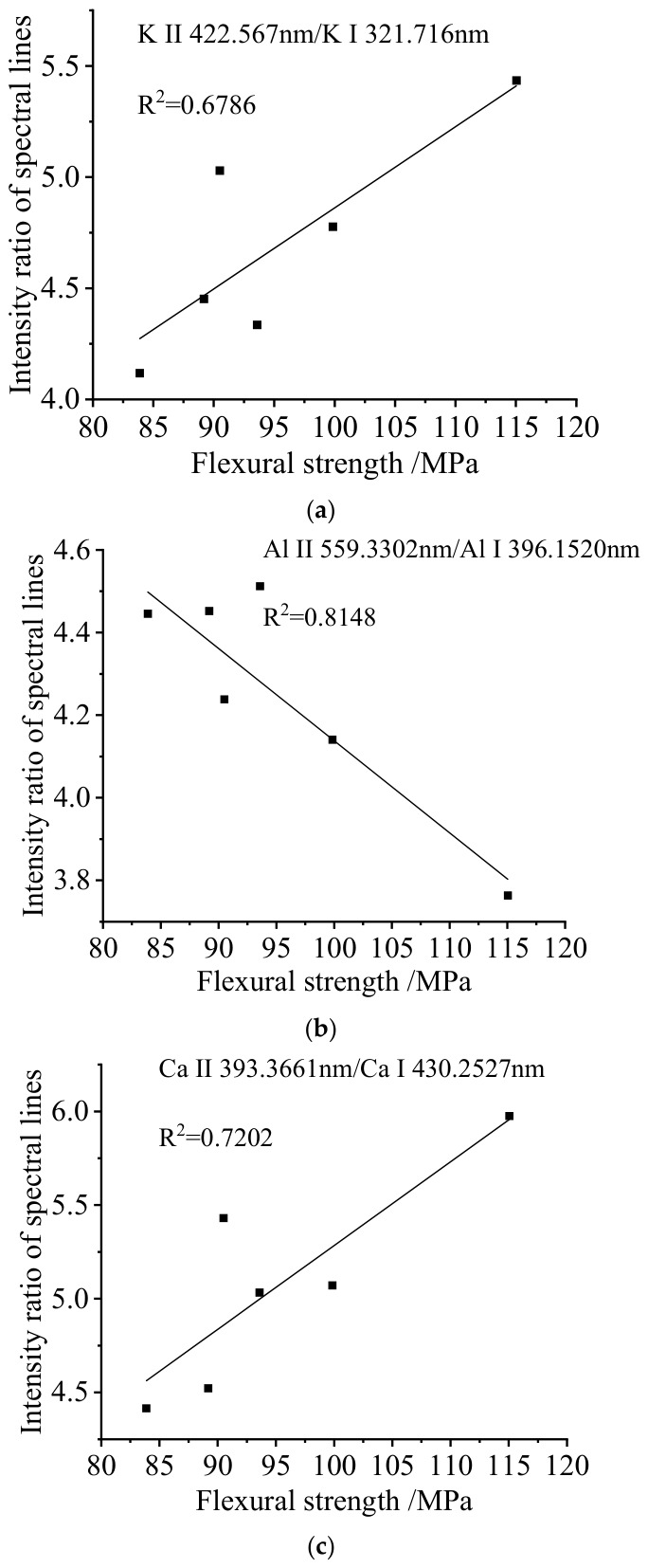
Relationship between SMC bending strength and different elemental ionic-to-atomic line intensity ratios: (**a**) K line ratio; (**b**) Al line ratio; (**c**) Ca line ratio.

In plasma characteristic analysis, the intensity ratio of ionic-to-atomic lines for different elements serves as a critical spectral parameter, directly reflecting the ionization equilibrium state within the plasma. This ratio complements plasma temperature diagnostics, jointly establishing a comprehensive parameter system for plasma state characterization [24,25,26,27]. Considering the elemental diversity in plasma composition, this study selected spectral lines of potassium (K), aluminum (Al), and calcium (Ca) to analyze bending strength characterization through ionic-to-atomic line intensity ratios. The analytical results are presented in Figure 15, which reveals varying degrees of linear correlation between SMC bending strength and elemental line ratios. The K line ratio (K II 422.567 nm/K I 321.716 nm) exhibits a positive correlation with bending strength (R^2^ = 0.6786), while the Al line ratio (Al II 559.3302 nm/Al I 396.1520 nm) shows a negative correlation (R^2^ = 0.8148). The Ca line ratio (Ca II 393.3661 nm/Ca I 430.2527 nm) demonstrates a positive correlation (R^2^ = 0.7202).

This experimental phenomenon originates from the modulation effect of SMC’s fiber–matrix composite microstructure on plasma excitation processes. Enhanced mechanical performance alters laser energy transfer mechanisms during ablation. Increased bending strength reduces plasma temperature, indicating suppressed plasma excitation that differentially affects elemental ionization levels. Variations in elemental ionization energies drive their distinct sensitivities to plasma parameters, explaining the opposing ionization trend observed for aluminum. The maximum R^2^ value of 0.8148 across all elemental correlations indicates the limited predictive capability of univariate linear models for bending strength quantification. This limitation arises from plasma fluctuations induced by SMC material heterogeneity, compounded by the complex influence of matrix–fiber interfacial element distributions on localized excitation conditions. While statistically significant correlations exist between line intensity ratios and bending strength, these parameters lack sufficient precision for quantitative mechanical property assessment.

### 3.4. Multivariate Linear Characterization of SMC Bending Strength

Previous research findings indicate that univariate linear models based on single-element characteristic spectral lines fail to achieve precise quantitative characterization of SMC bending strength. This limitation arises from the inherent deviations in spectral signal reproducibility caused by multiple factors influencing spatiotemporal evolution of laser-induced plasma, including excitation energy fluctuations, matrix effects, and environmental interference. When relying solely on a single spectral line intensity ratio as the input variable, transient plasma parameter variations significantly amplify data dispersion. This issue is exacerbated in heterogeneous composites like SMC, where differential laser energy absorption between fibers and the resin matrix further destabilizes plasma states. Consequently, univariate models exhibit low goodness-of-fit (R^2^ < 0.9) between elemental line ratios and bending strength, coupled with heightened sensitivity to experimental condition variations.

To address these challenges, this study developed a multivariate linear regression model integrating spectral line intensity ratios with plasma state parameters. The selected independent variables include plasma temperature ($T_{e}$), potassium line ratio (K II 422.567 nm/K I 321.716 nm, denoted $R_{K}$), aluminum line ratio (Al II 559.3302 nm/Al I 396.1520 nm, $R_{Al}$), and calcium line ratio (Ca II 393.3661 nm/Ca I 430.2527 nm, $R_{Ca}$)—all demonstrating measurable linear correlations with bending strength [28]. The resultant multivariate regression equation is expressed as:

(3) $$BS=a_{0}+a_{1}T_{e}+a_{2}R_{K}+a_{3}R_{Al}+a_{4}R_{Ca}+\varepsilon$$

where BS represents the dependent variable (bending strength), $a_{0}$~$a_{4}$ denote regression coefficients, and ε accounts for random errors.

The regression equation obtained through multivariate linear fitting is as follows:

(4) $$BS=472.6310-0.07894T_{e}-7.4372R_{K}-16.1516R_{Al}-3.2989R_{Ca}$$

**Figure 16:**
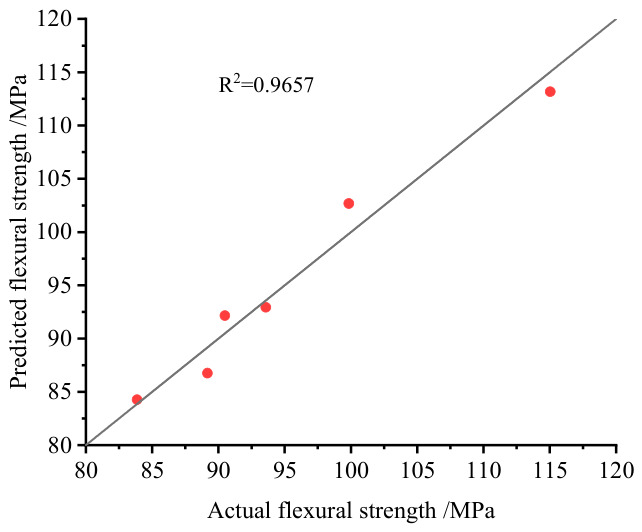
Fit results of the predicted values versus actual values using the multiple linear regression model.

Using this regression model, the predicted bending strength values for each group of SMC samples were calculated. The correlation between predicted and actual values is shown in Figure 16, with a goodness-of-fit R^2^ of 0.9657. These results indicate that the multivariate model, which integrates plasma temperature and characteristic line intensity ratios, shows better correlation with bending strength than the corresponding univariate models [29]. However, it should be noted that the present model was established based on a limited number of sample groups, and no independent validation set or cross-validation procedure was included in this study. Therefore, the proposed model should be regarded as a preliminary quantitative characterization framework, and its robustness and generalizability still need to be further validated using larger datasets and real field-aged samples in future work. In addition, factors such as self-absorption effects, plasma density variation, and experimental fluctuations may also influence the regression performance and deserve further investigation.

## 4. Conclusions

This study systematically investigated the characterization methods for the aging performance of SMC materials using laser-Induced Breakdown Spectroscopy (LIBS), elucidating the mechanisms of thermal aging effects on chemical structures and mechanical properties. A quantitative characterization model for SMC bending strength based on spectral data was further established. The primary research findings are as follows:

The thermal aging mechanisms and performance degradation patterns of SMC materials were clarified through multi-analytical approaches. Attenuated total reflection (ATR) infrared spectroscopy revealed the thermal decomposition of magnesium carboxylate salts and siloxane bond cleavage. Scanning electron microscopy (SEM) observations identified interfacial debonding between fibers and the resin, along with increased material porosity. ICP elemental analysis demonstrated 54.31%, 68.27%, and 56.56% loss rates for Ca, Al, and Si elements, respectively, uncovering synergistic mechanisms of filler depletion and interfacial deterioration.

A bending strength prediction model integrating multiple spectral features was developed. Initial univariate regression models correlating plasma temperature and elemental line intensity ratios (K, Al, Ca) with bending strength exhibited limited predictive accuracy (R^2^ < 0.9). Subsequently, a multivariate linear regression model incorporating all these parameters was constructed. The multivariate model achieved significantly improved prediction capability, with R^2^ between predicted and actual bending strength values reaching 0.9657, effectively addressing the precision limitations of single-variable spectral ratio predictions.

## Figures and Tables

**Table 1:** Bending strength of the samples.

Sample Number	Aging Time/h	Bending Strength/MPa
1	0	115.04
2	48	99.84
3	96	93.58
4	144	89.17
5	192	88.92
6	240	83.86

**Table 2:** Calcium (Ca) spectral lines used for constructing the Boltzmann plot.

Spectral Line	$\lambda_{ki}$ (nm)	$g_{k}$	$E_{k}$ (eV)	$A_{ki}$ (s-1)
Ca II	396.85	2	3.12	1.40 × 108
Ca I	422.67	3	2.93	2.18 × 108
Ca I	430.25	5	4.78	1.36 × 108
Ca I	430.77	1	4.76	1.99 × 108
Ca I	585.75	5	5.05	6.60 × 107

## Data Availability

The original contributions presented in this study are included in the article. Further inquiries can be directed to the corresponding author.
